# Characterization and Localization of Insoluble Organic Matrices Associated with Diatom Cell Walls: Insight into Their Roles during Cell Wall Formation

**DOI:** 10.1371/journal.pone.0061675

**Published:** 2013-04-23

**Authors:** Benoit Tesson, Mark Hildebrand

**Affiliations:** Marine Biology Research Division, Scripps Institution of Oceanography, University of California San Diego, La Jolla, California, United States of America; Iowa State University, United States of America

## Abstract

Organic components associated with diatom cell wall silica are important for the formation, integrity, and function of the cell wall. Polysaccharides are associated with the silica, however their localization, structure, and function remain poorly understood. We used imaging and biochemical approaches to describe in detail characteristics of insoluble organic components associated with the cell wall in 5 different diatom species. Results show that an insoluble organic matrix enriched in mannose, likely the diatotepum, is localized on the proximal surface of the silica cell wall. We did not identify any organic matrix embedded within the silica. We also identified a distinct material consisting of glucose polymer with variable localization depending on the species. In some species this component was directly involved in the morphogenesis of silica structure while in others it appeared to be only a structural component of the cell wall. A novel glucose-rich structure located between daughter cells during division was also identified. This work for the first time correlates the structure, composition, and localization of insoluble organic matrices associated with diatom cell walls. Additionally we identified a novel glucose polymer and characterized its role during silica structure formation.

## Introduction

Diatoms are one of the most ecologically successful microalgae and are important global primary producers. The reason for this success is a long standing question and has been the subject of numerous investigations. A distinct feature of diatoms is their cell wall called the frustule, which is made of silica and organic components. The cell wall consists of two halves, the hypotheca and epitheca; each theca consists of structures called valves, linked together by girdle bands which typically encircle the cell. The origin and the significance of this silicified exoskeleton remains unknown but it is thought to be an important part of diatom’s ecological success. Different roles have been attributed to the silica of the diatom cell wall, several of which could favorably affect diatom growth and productivity [1].

Silica structures of diatoms are formed inside the silica deposition vesicle or SDV [2], [3]. After completion, structures are exocytosed to form the cell wall. In general, organic components of the frustule vary in solubility or extractability and have been classified into three components, 1) an organic casing, which is a thin layer surrounding silica structures, 2) the diatotepum or diatotepic layer which is located between the plasmalemma and the silica, and 3) molecules or organic complexes trapped within the silica [4]. Additionally, mucilage is associated with the cell surface or secreted by diatoms. This mucilage can be used for motility, adhesion or protection against desiccation and predators [4].

Organic constituents trapped inside the silica have been characterized, including proteins called silaffins and silacidins, and long chain polyamines [5]. These molecules are soluble upon dissolution of the silica. They can self-associate in vitro and induce or control the polymerization of silicic acid into silica, however they don’t assemble into a solid matrix with higher order structure. The cytoskeleton is involved in higher order silica structure assembly, and actin in particular is thought to control the patterning of the silica from outside the SDV [6]–[9]. It is inferred from this that the SDV membrane serves as an interface to translate organizational information from extralumenal to intralumenal processes. Unfortunately, the composition of the SDV membrane as well as its role during silicification and its fate after exocytosis are unknown.

A long standing question has been about organic material associated with the frustule which could be involved in higher-order silica assembly. This was first observed intact and in cross section after dissolution of the silica with HF, forming a casing around the valves [10]. Von Stosch described a continuous organic layer only on the cell-proximal side of the silica (i.e. not a casing) between the plasmalemma and the frustule, which was named the diatotepum or diatotepic layer [11]. The diatotepum was proposed to hold the different parts of the frustule together and to also regulate the porosity of the cell [11]. The diatotepum was only observed after silica valves were exocytosed, suggesting that it was not directly involved in silicification, but added afterwards [12], [13]. It has been suggested that a polysaccharide matrix is embedded inside the silica and controls its morphogenesis [4], [14], [15]. Recently, Scheffel and coworkers isolated an insoluble organic matrix after SDS and HF treatment of purified cell walls [14]. The material, which mimicked the appearance of girdle bands in *T. pseudonana*, contained a family of proteins named cingulins. This matrix was shown to induce the polymerization of silicic acid in vitro in the presence of polyamines. Similar insoluble material that mimicked the valve structure of *T. pseudonana* was not identified, however such material was isolated from valves of *Coscinodiscus wailesii* [14]. A chitin-enriched organic network was observed after dissolution of the silica in *T. pseudonana*, which was suggested to be involved in valve morphogenesis [14].

In terms of polysaccharide components of the diatom cell wall, Hecky and coworkers [16] described organic components external to the silica in cell walls of different diatom species isolated by sonication, and identified the major sugar components as (in order of abundance) mannose, glucose and galactose. Sulphated glucuromannans (GMS) were first described by Ford and Percival [17] in cell walls of *Phaeodactylum tricornutum* and these were later found in *Navicula pelliculosa* and *Coscinodiscus nobilis* [18], [19]. McConville *et al* [20], using sequential extraction and transmission electron microscopy of *Stauroneis amphioxys* Gregory found glucuromannans in the insoluble fraction after alkali extraction at room temperature. However, transmission electron micrographs of cells after this treatment showed the presence of the silica structure and fibrillar material, which suggest that the low temperature alkali treatment didn’t completely solubilize the material. The data therefore may be consistent with material being located external to the silica. Volcani reported that in *P. tricornutum*, GMS is the end product of extraction and is localized at the proximal surface of the valve [19]. More recently Chiovitti *et al* characterized polysaccharides associated with silica in the cell wall in different diatom species [15]. The most abundant silica-associated sugar found for the 4 different species analyzed was mannose. Further analysis showed that the mannans were composed mostly of 3-linked mannans which were polyanionic with high levels of uronic acid and/or sulfate esters. The authors suggested that the mannan-enriched material was embedded within the silica and might have a role during its morphogenesis.

Another insoluble polysaccharide associated with the frustule is callose, which is a polymer of β-1, 3-glucose. Waterkein and Bienfait localized this component associated with different parts of the cell wall of *Pinnularia* using Aniline blue [21]. It was found at three particular locations in the cell wall, 1) between the hypotheca and the epitheca, 2) as two strips called the “nebenlinien” associated with the cingulum and, 3) transiently associated with the newly formed valves during division. These callosic deposits were interpreted as joints protecting the cells from the surrounding medium. Since this initial investigation, no further work on the role and localization of callose in different diatom species has been performed.

It is clear from the literature that there is a diversity of types of organic material associated with diatom cell walls, but definitive understanding of their roles is still lacking. Previous studies characterized the biochemical nature of organic components of the cell wall, however, information about the localization and the physical nature of these components are still lacking. A critical aspect of characterization, especially in terms of localization, is the extraction procedure. We summarize the different extraction approaches that have been applied in Fig. S1. In all previously-mentioned studies, mannans were found in the hot alkali fraction after dissolution of the silica. However since the methods applied were not sufficient to remove all external organics, their localization inside or outside of the silica cannot be stated with certainty. Determination of the location of these materials relative to the silica would greatly aid in understanding their functions.

In this study we first isolate and localize insoluble cell wall-associated organic material and then determine its composition. We examined seven diatom species to evaluate differences between species, and provide a comparative characterization of the insoluble organic material, including a description of structure, mechanical properties, localization, and monosaccharide composition. Additionally we identified a novel glucose polymer and characterized its role during silica structure formation.

## Materials and Methods

### Diatom Species and Culture Conditions

*Amphora salina* (CCMP1119), *Nitzshia curvilineata* (CCMP555), *Stephanopyxis turris* (CCMP815), *Coscinodiscus radiatus* (CCMP310), *Navicula cryptocephala* (CCMP2519) and *Triceratium dubium* (CCMP147) were obtained from the Provasoli-Guillard National Center for Culture of Marine Phytoplankton, Bigelow Laboratory for Ocean Sciences (West Boothbay Harbor, ME, USA). Diatoms were grown in ASW [22] medium under bubbling with air under continuous light at 19°C. In order to enrich the culture in cells making valves, cultures were synchronized as described previously [23]. For the treatment with β-1, 3-glucose synthase inhibitor, solution of 15 mM of mycafungin (Mycamine, astellas Pharma, US) was prepared in milliQ water and inoculated into the culture medium at various concentration between 5–200 µM.

### Preparation of the Insoluble Organic Matrix

Diatoms were harvested by centrifugation at 4000 g, cleaned three to four times by treatment with 2% SDS, 0.1 M EDTA at 95°C for 30 min followed by centrifugation, rinsed once in milliQ water, washed for 45 min in acetone and rinsed 3 times in milliQ water. Then the silica was dissolved in a mixture of HF/NH_4_F (2 M/8 M) at pH 4–5 for 15 min, centrifuged at 10,000 g and rinsed 4 times in milliQ water. To remove all external organic material before silica dissolution, hydrochloric acid and hydrogen peroxide treatments were applied. After SDS treatment, samples were boiled three times in 6 N HCl or 30% H_2_O_2_ for 40 min and rinsed 3 times with milliQ water.

### Sample Preparation for Fluorescence Microscopy

Silica structure was visualized by addition in the culture medium of 100 ng · mL^−1^ of LysoTracker Yellow HCK-123 [24]. Calcofluor, DAPI and aniline blue (Sigma Aldrich) were used at a final concentration of 0.01%, 2 µM and 0.05% respectively. Samples were imaged using a Zeiss AxioObserver inverted microscope equipped with an Apotome (Carl Zeiss Microimaging, Inc., Thornwood, NY, USA). The filter set used for calcofluor, aniline blue and DAPI was Zeiss #21HE (Ex 387/15 nm, FT 409, Em 510/90 nm) and for LysoTracker Yellow HCK-123, Zeiss #38HE (Ex 470/40 nm, FT 495 nm, Em 525/50 nm), respectively. With these filters and under the exposures used, no chlorophyll autofluorescence was visible. Chlorophyll was imaged using Zeiss filter set #16 (Ex 485/20 nm, FT 510 nm, Em 515 nm LP). Images were acquired with 63×/1.4 objective oil immersion plan APO and treated using Axiovision 4.7.2 software.

### Preparation of Frustules for SEM Examination

For removal of external organic material, diatom frustules were cleaned by acid treatment. Ten milliliters of diatom culture was harvested by centrifugation at 3,000×g for 4 min and frozen at −20°C. Cells were boiled in 400 µl concentrated sulfuric acid for 10 min, cooled, then 20 mg KNO_3_ was added, followed by boiling an additional 10 min. Frustules were then washed using centrifugation three times with ultrapure water. For SEM examination, samples were sputter coated with iridium and observed with an FEI SFEG UHR (FEI Company, Hillsboro, OR, USA) scanning electron microscope at the University of California San Diego Nano3 Facility.

### Atomic Force Microscopy

A Veeco Bioscope catalyst Atomic Force Microscope coupled with a Zeiss inverted fluorescent microscope was used for imaging and elasticity mapping. RTESP and TAP525 (Bruker) were used for imaging and TAP525 was used for elasticity measurement. Imaging was realized in Scanasyst mode. The peak force Quantitative Nanomechanical Mapping (PFQNM) mode was used for the generation of the elastic modulus map. PFQNM is a new AFM mode developed by Bruker that enables the extraction of mechanical properties at high speed and high resolution [25].The relative method was used for calibration of the probe. Deflection sensitivity was determined on a sapphire surface, the tip radius and spring constant were calibrated respectively on the titanium oxide and polystyrene surface (2.7 GPa).

### Monosaccharide Analysis

The purified organic matrices were analyzed for monosaccharide content. Methanolysis of the samples was carried out using 1 M MeOH-HCl at 80°C for 18 h, followed by removal of the acid and re-N acetylation using methanol: pyridine: acetic anhydride in 4∶1:1 ratio at 100°C for 1 h. Removal of the reagents and trimethylsilyl (TMS) derivatization was realized using Tri-Sil reagent at 80°C for 30 min. Finally the samples were dissolved in Hexane and run on GC-MS.

## Results

In order to isolate the insoluble organic matrix associated with the cell wall of different diatom species, we first boiled the cells in SDS to remove intracellular and soluble cell wall-associated material and then dissolved the silica with a mixture of HF/NH_4_F (Fig. S1). The remaining components had the shape and structure of the valve and girdle bands. We analyzed and compared the structure, mechanical properties and composition of these matrices in five different species.

### Structure of the Organic Matrices Associated with the Frustule

**Figure 1:**
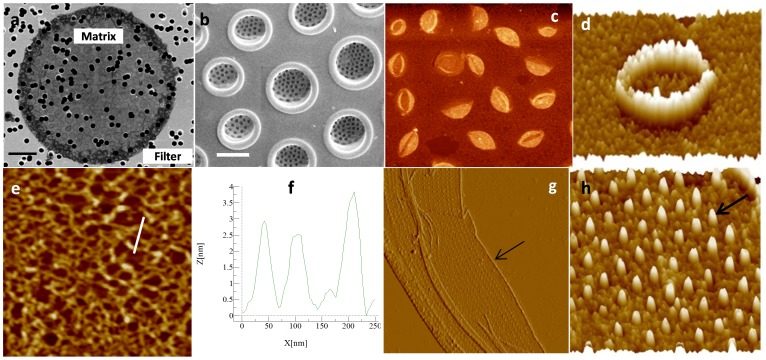
*C. radiatus* silica structure and organic matrix. a; SEM micrograph of the valve matrix deposited on a filter (scale bar 5 µm). b; SEM micrograph of the proximal surface of the valve (scale bar 1 µm). c–e; AFM micrographs of the organic matrix associated with the valves, c) shows the organization of material corresponding to the foramen, d) shows a high resolution image of material corresponding to a single foramen, e) shows the fibrous nature of the material between foramen, and f) shows a height profile of the line in e. g and h; organic material associated with the girdle bands. The arrow in g points at the higher edge and in h shows nodules filling the pores in the silica structure (scan size for AFM images: 9.3x8.1, 2, 1.2, 10 and 2 µm respectively).

In *C. radiatus*, the matrix was the full diameter of the valve (Fig. 1a) and contained round structures regularly arranged and corresponding in size to the foramen opening (Fig. 1a–d). The surface of the material appeared rough, it was composed of fibers approximately 50 nm wide and 2.5 to 4 nm high (Fig. 1e and f). The organic material was approximately 10 nm thick. The insoluble organic matrix associated with the girdle bands was covered with 100 nm wide and 70 nm high nodules, corresponding to the openings of pores in the girdle bands (Fig. 1g and h). This suggested that the pores are not completely open, but are lined with organic material. The periphery of the matrix corresponding to the edges of the girdle bands that do not display pores was free of nodules. This was also a region of overlap between girdle bands, and the extreme edge was seen to have a thicker structure (arrows in Fig. 1g).

**Figure 2:**
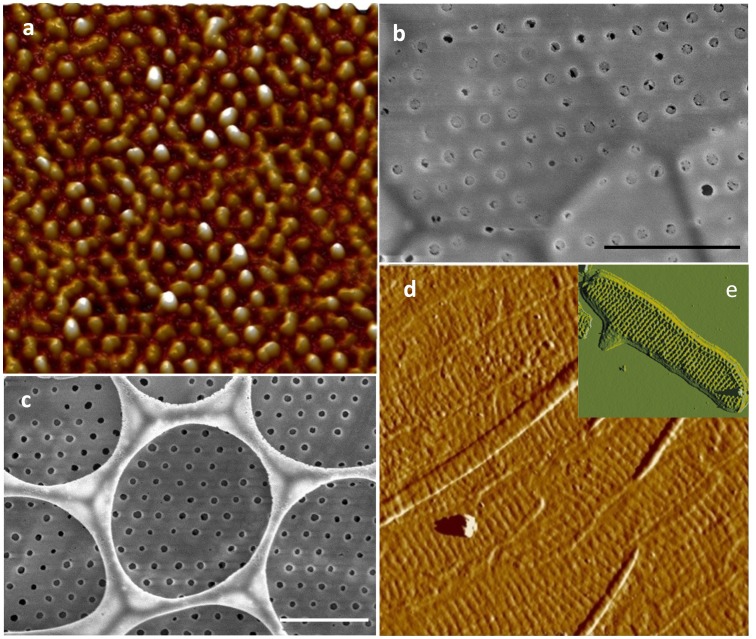
*S. turris* silica structure and organic matrix. a and d; AFM micrographs of the organic matrix associated with the valves and the girdle bands respectively. b and c; SEM micrographs of the proximal and distal surface of the valve respectively (scale bar 1 µm). e; AFM micrograph of a silicified girdle band. AFM scan sizes are 3.6, 10, and 10.6 µm for a, d, and e respectively).

In *S. turris,* the matrix from the valve was covered with nodules (Fig. 2a) corresponding in spacing and hexagonal arrangement to the pores present in the proximal surface (Fig. 2a and b). The organic material was not fibrous at the nanoscale (Fig. 2a). No pattern corresponding to the large hexagonally-arranged ribs on the distal surface of the valves was observed, suggesting that the matrix was localized only on the proximal surface (Fig. 2a and c). The girdle bands of *S. turris* look like scales (Fig. 2e) and the matrix associated with them consisted of an assembly of scale-shaped structures (corresponding to individual girdle bands) that maintain the striations corresponding to the silica ribs of the girdle bands (Fig. 2d).

**Figure 3:**
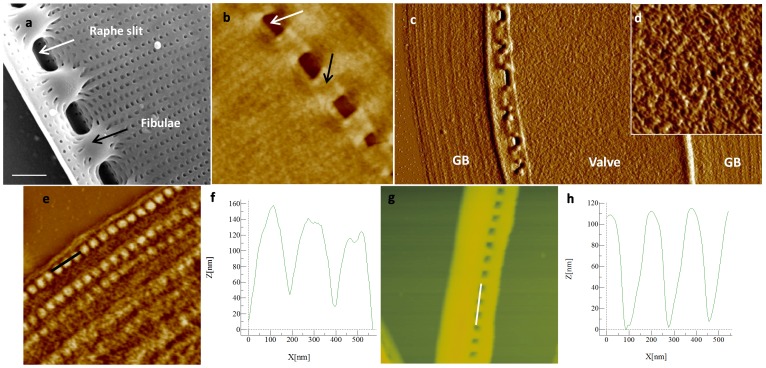
*N. curvilineata* silica structure and organic matrix. a; SEM micrograph of the proximal side of the valve (scale bar 1 µm). b–h; AFM micrographs and height profiles. Arrows in a and b show the fibulae (black) and the location of the raphe slit (white). b and d; Organic matrix associated with the valves. c; organic matrix associated with the valve and girdle bands (GB). e; organic matrix associated with the GB and corresponding height profile (F). g; Silicified GB and corresponding profile (h). AFM scan sizes are 5, 20×9, 2, 3.1 and 2.7 µm for b–e and g, respectively).

In *N. curvilineata*, the valves contain particular structures called the fibulae that attach the two parts of the valve together and enclose the raphe slit (Fig. 3a). The organic matrix covered the entire valve including the fibulae, however the raphe slit remained unobstructed (arrows, Fig. 3a and b). We also observed undulations on the matrix surface in contact with the valve corresponding to the silica ribs (Fig. 3a and b). In this species, we observed a continuous insoluble matrix that included the patterns of the valve and the girdle bands (Fig. 3c). At the nanoscale, the valve-associated organic material was highly textured and seemed to be made of the assembly of fibrous structures with some similarities to *C. radiatus*, however it appeared more dense and intricate, which might be due to its greater thickness which was about 100 nm (Fig. 3d). The organic matrix associated with the girdle bands (Fig. 3e) contained nodules which correspond in size and spacing to the pores in the girdle band silica (Fig. 3g) as shown by the height profiles on the AFM image of the silica structure and the matrix (Fig. 3f and g).

**Figure 4:**
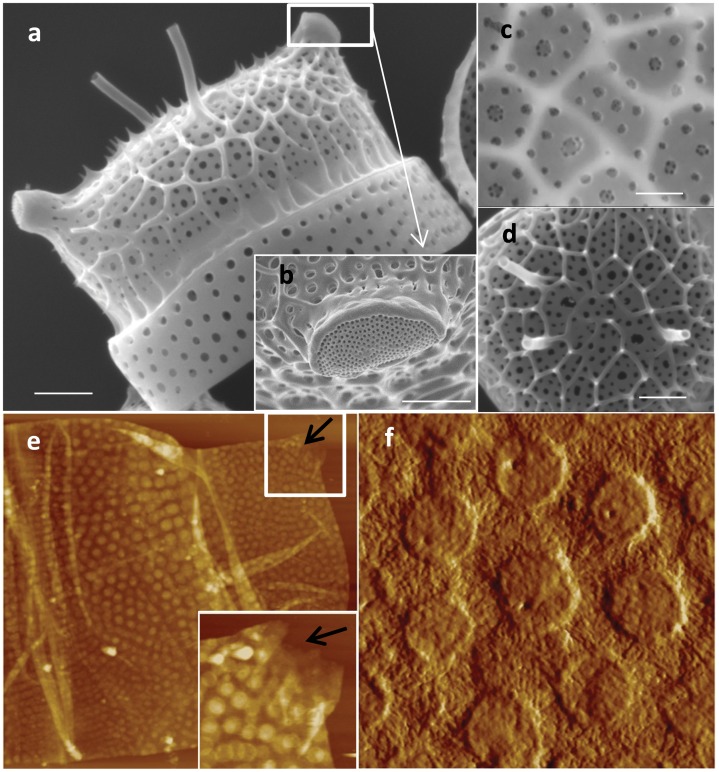
*T. dubium* silica structure and organic matrix. a–d; SEM micrographs of the valve structure. b; ocellus, c; proximal surface. d; distal surface. e and f; organic matrix associated with the valves and girdle bands. e inset; zoom on the ocellus opening, the arrow show the opening in the ocellus. AFM scan sizes, 24, 3.5 and 2 µm for e, inset e, and f, respectively, scale bars a, b and d = 2 µm, c = 1 µm.

In *T. dubium* the valves contained diverse substructures such as spines, and perforated dome shaped structures called ocelli, which enable secretion of substances into the medium (Fig. 4a–d, [26]). The valves are made of interconnected silica ribs on the distal side, with perforated silica sheets on the proximal side, similar to *S. turris* (Fig. 4c and d). Insoluble organic material from *T. dubium* showed heterogeneity at the nanoscale with the presence of relatively smooth round structures interspersed with fibrous material (Fig. 4e and f). The round structures correspond in position and size with the pores on the proximal valve surface, consistent with the organic layer being present on that side. Correspondingly, there was no evidence of the larger silica ribs characteristic of the distal valve surface in the material. Interestingly, the organic layer was absent underneath the ocelli (arrow in Fig. 4e). Like the raphe slit, this particular substructure is involved in secretion of mucilage, and the absence of organic layer underneath this structure suggests an arrangement that facilitates secretion of material into the medium.

**Figure 5:**
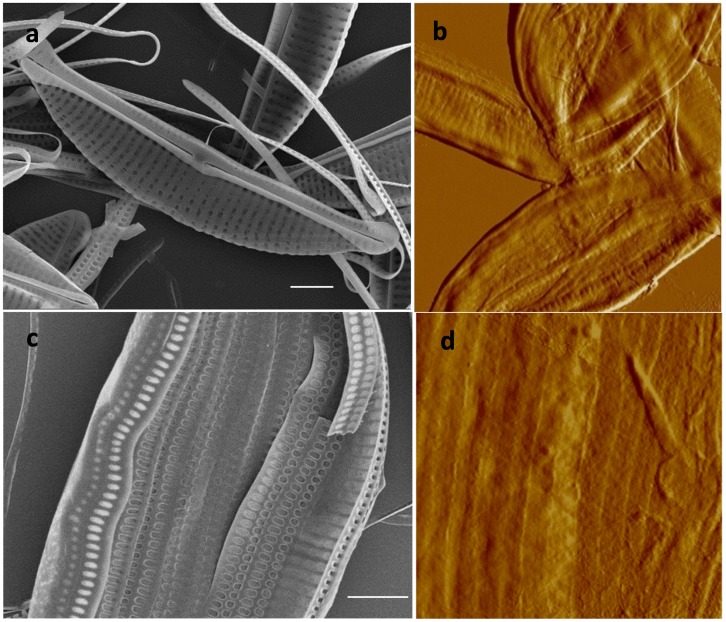
*A. salina* silica structure and organic matrix. a and c; SEM micrographs of the valve and girdle bands respectively (scale bar 2 µm). b and d; AFM micrographs of the organic matrix associated with the valves and the girdle bands respectively (scan size: 15 and 5 µm respectively).

The organic layer associated with the *A. salina* frustule was relatively smooth. On the valve, some appearance of ribs was visible (Fig. 5a and b). Longitudinal lines which seem to correspond to the separation between girdle bands were readily visible (Fig. 5c and d).

All extracted insoluble organic material described so far could be stained with DAPI (examples in Fig. S2 and S3), consistent with a poly-anionic composition. DAPI is known to stain DNA, polyphosphate and poly-anionic structures and has been observed staining the forming cell wall during diatom division [27]. Interestingly, we did not observe DAPI staining of cell walls in intact cells, suggesting that the material was inaccessible. One possible explanation is that removal by SDS of an additional layer covering the matrix was required to make it accessible.

### Localization of the Matrix

**Figure 6:**
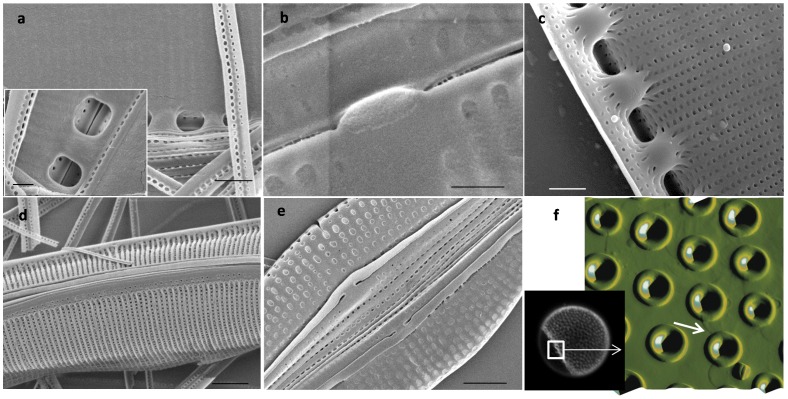
Localization of the organic matrix in *N. curvilineta* (a, c and d), *A. salina* (b and e) and *C. radiatus* (f). a, b, d and e: SEM of SDS-cleaned valves. a and b: SEM of valve proximal surface showing the organic matrix. d and e: SEM of distal surface showing naked silica. c: SEM of acid cleaned valve proximal surface (compare with a). Scale bar: a, c = 1 µm, d, e = 2 µm, a inset, b = 500 nm. f and inset; AFM and fluorescent micrographs of DAPI-stained SDS-cleaned *C. radiatus* valve showing the area where the organic layer peeled off (arrow in f).

The consistent correlation in patterning and features suggested that the insoluble organic matrix formed a layer on the proximal valve surface. To substantiate this, we compared cells treated with SDS with those treated with sulfuric acid by SEM (Fig. 6). In SDS-cleaned *N. curvilineata* and *A. salina* the organic layer was present on the inner surface of the frustule where it obscured the silica structure (Fig. 6a and b). No organic material was observable on the distal surface of SDS cleaned frustules (Fig. 6d and e) or on the proximal surface of acid cleaned valves (Fig. 6c – compare with a).

In *C. radiatus*, the matrix was so thin that it wasn’t visible by SEM even at low voltage. Using DAPI to stain the matrix, we observed it peeling off the valve after SDS treatment (Fig. 6f). By AFM we were able to confirm the localization of this layer on the proximal surface of the wall. The round structures observed in the matrix correspond to the area surrounding the opening of the foramen and covering the inside of the cribrum (Fig. 1b and d, 6f). The organic layer was also observed peeling off the cell wall of *S. turris* (not shown), however in the case of *N. curvilineata* and *A. salina* it seemed to be more tightly associated with the silica.

To evaluate whether an insoluble matrix was embedded within the silica, we first treated *N. curvilineata* and *T. dubium* with hydrochloric acid to degrade organic material external to the silica. It was previously shown that such treatments do not affect organic components embedded within the silica [28], [29]. After dissolution of the silica with HF following this treatment, we observed no structured organic material, confirming the absence of an insoluble organic matrix trapped inside the silica structure (data not shown).

### Structure and Localization of a Glucose Polymer

**Figure 7:**
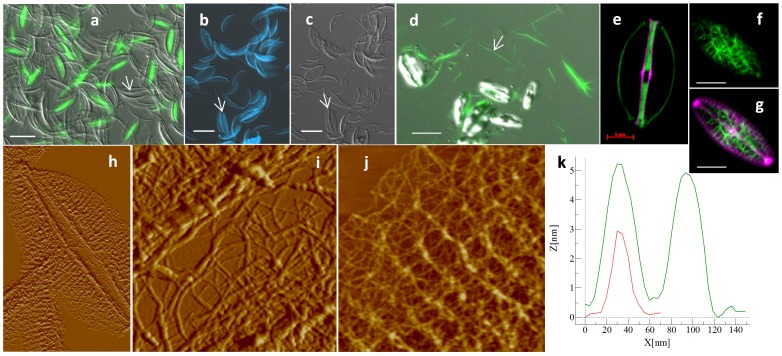
a–c; SDS cleaned cell wall of ***A. salina***. a; DIC and calcofluor (green fluorescence), b and c; DAPI (blue fluorescence) and corresponding DIC micrographs. Arrows show silica valve structures. d; *A. salina* in DIC and stained with calcofluor, arrow shows the fibrillar structure released into the medium. e; *A. salina* stained with calcofluor (green) and HCK123 (pink). f and g; *N. cryptocephala* stained with calcofluor and HCK123. h–j; AFM micrographs of the calcofluor stainable organic structure. h; height profile of single fibril from j. AFM scan sizes, 16.4×10.2, 1.7 and 2.8 µm for h–I, respectively, scale bars; a–d = 20 µm, e–f = 5 µm.

In *A. salina*, additionally to the insoluble matrix associated with the frustule, we observed a distinct organic structure which stained with calcofluor (Fig. 7a) In SDS-cleaned frustules, DAPI stained the previously-described matrix, which was associated with cell wall silica (Fig. 7 b and c). In intact cells a calcofluor-stained structure was observed between daughter cells and also free in the medium (Fig. 7d and e). This structure was present during cell division and localized between the two protoplasts during silica formation (Fig. 7e). We assume that after separation of the daughter cells it was released into the medium (Fig. 7d and e). We observed similar material in another pennate diatom, *Navicula cryptocephala*, which in valve view exhibited a network appearance (Fig. 7f and g). Using AFM to characterize the calcofluor-stained material from *A. salina* indicated that it consists of two halves connected by fibers, each half has the shape and the size of a valve (Fig. 7h). The fibers were organized as a dense network forming a thick bundle at the interface between the two halves (Fig. 7h–j), striations were observable perpendicularly to the bundle and corresponding to the organization of the silica ribs of the valve (Fig. 7j). The fibrils in this material were 30 to 50 nm wide and 3 to 5 nm high (Fig. 7k).

**Figure 8:**
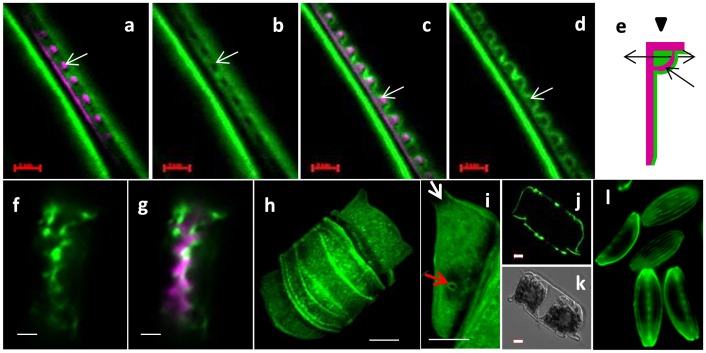
a–g; Fluorescent micrographs of whole cells stained with calcofluor (green) and HCK123 (pink). a–d; Two different optical sections (a, b and c, d) of dividing *N. curvilineata* in longitudinal section (scale bars 2 µm). e; sketch of *N. curvilineata* valve in transversal section showing the orientation of the section (double arrow) and the view (arrow head at top) of a–d. White arrows in a-d show a fibulae surrounded by calcofluor stained material, and correspond to the dark single-headed arrow in e. f and g; Longitudinal section of dividing *C. cryptica* (scale bars 2 µm). h–k; *T. dubium* stained with calcofluor (scale bars 5 µm). h; reconstructed 3D image of whole cell. i; Zoom on the matrix associated with the valve showing the absence of matrix in the area corresponding to the ocelli (white arrow) and the spine (red arrow). j and k; optical section through a dividing cell, calcofluor (j) and corresponding DIC (k). l; *A. salina* stained with calcofluor (scale bar 5 µm).

Calcofluor stained the cell wall of other diatom species, however the location of staining was not conserved (Fig. 8). In *N. curvilineata*, during valve formation, calcofluor stained a material covering the inner surface of the valve (Fig. 8a–d), rather than between the cells as in *A. salina*. The location of this structure in *N. curvilineata* differed slightly from the matrix imaged by AFM, indeed the calcofluor-stained structure surrounded the fibulae and covered the raphe slit (arrows in Fig. 8a–e), whereas the matrix layer was not present over the raphe slit (Fig. 3b and 6a). This structure was found during valve formation but also in some mature valves (not shown), suggesting that it remains associated with the valve for some time and eventually is degraded or hidden by the deposition of additional material. In *C. cryptica*, a calcofluor-stained structure was also observed on the proximal surface following the contour of the forming valves (Fig. 8f and g). In *T. dubium,* calcofluor and DAPI both stained organic material associated with the entire frustule (Fig. 8h–k and Fig. S3). The absence of fluorescence in the area underneath the ocelli and at the base of the spines confirmed the observation of lack of organic material by AFM (Fig. 4e and 8i). We didn’t observe calcofluor fluorescence associated with forming valves in this species, suggesting that the material was added later during maturation of the wall (Fig. 8j and k). Calcofluor-stained regions were also observed between girdle bands in *A. salina, N. curvilineata* and *C. radiatus* (Fig. 8l and Fig. S4).

### Mechanical Properties and Composition of the Insoluble Polymers

**Figure 9:**
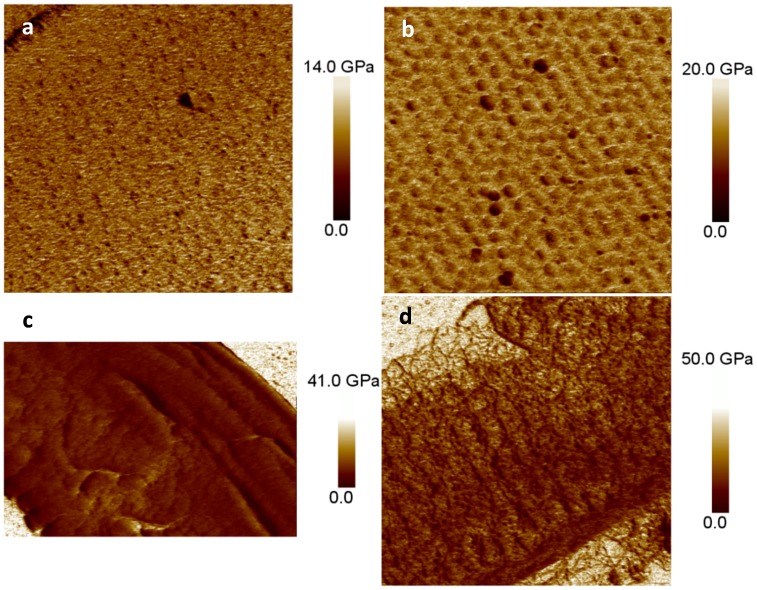
Peak Force Quantitative Nanomechanical (PF QMN) mapping. The elasticity of the organic matrix associated with *N. curvilineata* (a), *S. turris* (b), *A. salina* (c), and calcofluor-stained material from *A. salina* (d) are shown.PF QNM generates an elasticity map over the entire scanned region, variations in intensity relate to differences in elasticity. Mean elasticity value and scans size are respectively; 7.1, 11.6, 6.8 and 10.7 GPa and 5, 3.6, 5×3 and 5 µm.

In order to assess the mechanical properties of the organic matrices, we used peak force quantitative nanomechanical mapping (see Methods) to map the elastic modulus. We compared the insoluble organic matrix (DAPI stained) from *N. curvilineata, S. turris* and *A. salina*, and the calcofluor-stained material from *A. salina*. The mean values measured for the DAPI-stained material from valves ranged from 7.1 GPa (*N. curvilineata*), to 11.6 GPa (*S. turris)*, to 6.8 GPa (*A. salina*), and the calcofluor-stained material from *A. salina* was 10.7 GPa (Fig. 9). The mechanical properties were relatively homogeneous across all of the material regardless of any surface structure, suggesting that no specialized material was used to make particular features.

Although we could image and map the mechanical properties of the organic matrices distinctly, we could not physically separate the DAPI and calcofluor-stained material for sugar analysis, but instead subjected the entire organic extract to this approach. The organic material in *C. radiatus* was mainly constituted (Table 1) of mannose (80.1%) and glucuronic acid (12.4%). In *A. salina* and *N. curvilineata* we found a higher proportion of glucose (47.7 and 45.6% respectively). In *T. dubium*, the amount of glucose was relatively low (6.4%), however we found 12.8% of N acetyl-glucosamine. The other sugars present were rhamnose, xylose, galactose and galacturonic acid (Table 1). These results confirm that glucuromannans, the final product of sequential extraction found in every diatom species studied thus far, are found in the organic materials described in this study. Additionally the higher amount of glucose found in *A. salina* and *N. curvilineata* and the N acetyl-glucosamine found in *T. dubium*, confirms the staining observation made with calcofluor, and suggests that this material has a composition distinct from that of the DAPI-stained matrix (Table 1).

**Table 1:** Monosaccharide composition (mol %) of the organic matrix and localization of stained structure (calco = calcofluor).

	Rha	Xyl	Man	Glc	Gal	GlcA	GalA	NAcGlc	Matrix	Forming Valves	Betweencell	Between GB
***C. radiates***	1.5	1.4	80.1	4.7	tr	12.4	tr	–	DAPI	–	–	Calco
***N. curvilineata***	1.7	4.4	40.3	45.6	6.5	1.4	–	–	DAPI	Calco	–	Calco
***A. salina***	1.5	1.3	41.5	47.7	2.7	5.5	tr	tr	DAPI	–	Calco*	Calco
***T. dubium***	tr	6.3	67.4	6.4	tr	1.5	5.6	12.8	DAPI/Calco	–	–	Calco

* Described in fig. 8.

Rha : rhamnose, Xyl : xylose, Man : mannose, Glc : glucose, GlcA : glucuronique acid, GalA : Galacturonique acid, NAcGlc : NacetylGlucosamine.

By looking at the composition and organization of the organic matrices associated with the different diatom species studied and more specifically at the calcofluor staining behavior, we could separate them into distinct groups (Fig. S6). One group included *S. turris* and *C. radiatus*, which did not show any calcofluor stained structure associated with their valves. A second group included *T. dubium* which had an organic matrix corresponding to the entire frustule which was stained both by DAPI and calcofluor. This species also had the unique presence of N acetyl-glucosamine. A third and fourth group included *N. curvilineata*, *N. cryptocephala* and *A. salina* which had calcofluor-stained structures associated with their valves only during cell division. In *A. salina* and *N. cryptocephala*, calcofluor staining was seen between daughter cell protoplasts during valve formation, while in *N. curvilineata*, it occurred on the proximal valve surface (Fig. S6). In contrast to the variable appearance and location of the calcofluor-stained material, all species contained DAPI-stained material after SDS treatment that mimicked the structure of the proximal surface of the valve or girdle band.

### Role of the Glucose Polymer

**Figure 10:**
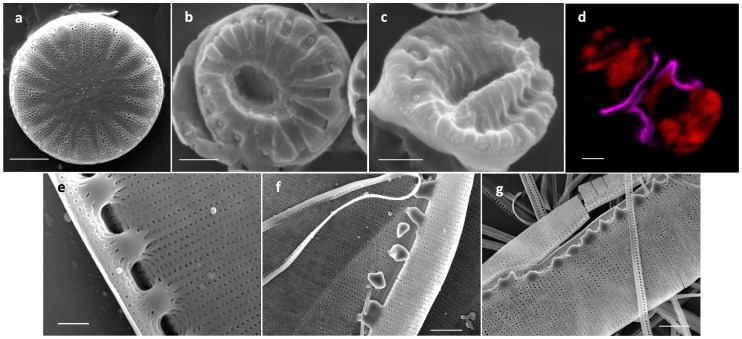
The effect of mycafungin (a specific β-1-3-glucan synthase inhibitor) treatment on valve formation. a–c, b–g; SEM micrographs of acid cleaned frustules of *C. cryptica* and *N. curvilineata*. a and e; controls. b, c and f, g; Treated with mycafungin. d; Fluorescent micrograph of HCK123 (pink)-stained *C. cryptica* treated with mycafungin (red = chlorophyll autofluorescence). Scale bar: a–d and f, g = 2 µm, e = 1 µm.

In diatoms, glucose polymers are mainly β-1, 3-glucose, and called chrysolaminarin for the storage form and callose for the fibrillar form associated with the cell wall [21]. In order to clarify the role of callose during cell wall synthesis, we decided to test the effect of Mycafungin, (Fig. 10). Mycafungin is an echinocandin antifungal drug which inhibits β-1-3-glucan synthase specifically [30]. It was tested on 3 species, *N. curvilineata*, *C cryptica* and *A.* salina, in which calcofluor staining was observed during valve formation. In *C. cryptica*, cell division was almost completely arrested at a concentration of 20 µM and highly reduced at 5 and 10 µM. After a few days of growth in the presence of 5 and 10 µM of mycafungin, the silica structure of the valve of *C. cryptica* was dramatically affected at the microscale. The overall shape of the valve appeared disorganized (Fig.10a–c). In order to check if this was due to disfiguration of the silica structure during sample preparation because of an effect by the drug on the amount of silica deposited, we observed the valve structure in vivo after staining with HCK123. Results show that the structure of the valve was affected similarly, which means that the observed effects were due to an alteration of the mechanism of morphogenesis at the microscale (Fig. 10d). In *N. curvilineata*, we observed an effect on growth rate starting at 5 µM mycafungin. At 10 and 20 µM, the growth rate was highly reduced and we observed defects in the valve structure (Fig. 10e–g). The fibulae were particularly affected, sometimes failing to attach the two parts of the valve together, which corroborates with the calcofluor localization (Fig. 8a–e and 10e–g). In *A. salina* growth was highly reduced at 50 µM, however at this concentration and higher (80 µM) we didn’t observe any noticeable effect on the cell wall structure.

## Discussion

### Comparison with Material Identified in Other Studies

A critical aspect of characterization of diatom cell wall-associated organic material has been the procedure used for isolation (Fig. S1). Unless all organic material external to the silica is degraded prior to silica dissolution, it cannot be conclusively said that the material is embedded within the silica. Chiovitti and coworkers described mannans associated with the frustule of 4 different diatom species [15]. The polysaccharides were extracted from the detergent-treated frustules using hot alkali to dissolve the silica, and the authors concluded that they were encapsulated within the silica. In this study we used imaging approaches to show that mannans are localized on the inner surface of the frustule even after detergent treatment – tightly associated but not integrated within it. Brunner and coworkers found a chitin network associated with the valves of *T. pseudonana* and suggested that this material might be involved in the morphogenesis of the valves [31]. In this study the only place we found N acetyl-glucosamine was in the *T. dubium* organic matrix which was associated only with the mature cell wall suggesting that it is not involved in silica formation. The cingulin-associated insoluble organic material characterized from *T. pseudonana* by Scheffel *et al*, was proposed to be embedded within the silica of the girdle bands, however the isolation procedure used could not distinguish between that or a surface-association with the silica [14]. We performed SEM characterization of SDS-cleaned material from *T. pseudonana*, which suggested an association of organic material with the inner surface of the girdle bands (Fig. S5).

After complete dissolution of SDS-cleaned silica, we observed an insoluble matrix which we correlated by SEM and AFM to be located on the proximal surface of silica structures. The lack of material other than this is consistent with the absence of an additional matrix embedded within the silica. To confirm this, we first cleaned *N. curvilineata* and *T. dubium* silica with either HCl or H_2_O_2_, followed by silica dissolution by HF. There was no visible organic material present in the acid-treated samples, whereas organic material corresponding to the matrix was present in the H_2_O_2_-treated samples (data not shown). These experiments confirmed the absence of insoluble organic matrix trapped inside the silica.

### Role of the Mannan-rich Organic Matrix

The association of this organic matrix with the proximal surface of the frustule is consistent with the matrix corresponding to the diatotepum as described by Von Stosch [11]. A question that is raised by our results and those of Scheffel *et al* is whether or not the organic matrix is involved in silicification [14].

TEM studies [12], [13] described the diatotepum as being added after exocytosis of the valves, which suggests that it does not play an active role in silicification. However, it could have been present during valve formation but not visible by TEM because at that stage the layer was thin and was tightly associated with the silica and/or the silicalemma.

There are several pieces of evidence consistent with the organic matrix being involved in silica structure formation. The exact correlation between silica structure and the appearance of the organic layer suggests an intimate association in time and space. The organic matrix remains associated with the frustule even after boiling in SDS, indicating a strong interaction between these two components. The matrix is made of the assembly of parts which are the shape of distinctly-synthesized frustule substructures (i.e. girdles bands and valves), instead of being a single continuous layer underlying the entire frustule as previously suggested [20], although some of these substructures do remain attached together after extraction (Fig. 2d and 3c). The presence of nodules in the organic material that correspond to nanoscale pores in the silica (Figs. 1h, 2a, 3e) also indicates an intimate interaction with the silica. Although there may be a mechanism to enable extrusion of the organic material after the pores are made, given the pores small size (<50 nm) it seems equally if not more plausible that the material was present initially and the silica polymerized around it to form the pore. Observations on the formation of 20 nm pores in the valve of *T. pseudonana* are consistent with the presence of material preventing polymerizing silica from filling in the space that eventually becomes a mature pore [32]. There is a tendency in diatoms for initial silica deposition to form a base layer of the proximal surface, followed by z-axis growth towards the distal surface [33]. Proximal structures tend to be more highly ordered than distal ones [34], which implies that the organic material controlling structure is associated with, or influences that, surface more. The organic material described by Scheffel *et al* is likely to correspond to the matrix described in this study, in which case, its silica precipitation activity in the presence of polyamines would be consistent with a role in silica formation in vivo [14].

### Role and Significance of the Glucose Polymer Associated with the Frustule

We also found glucans associated with the cell wall of diatoms. Waterkeyn and Bienfait, using aniline blue staining, found callosic structures (β-1, 3-glucose) localized between the theca, associated with the cingulum and transiently with the valves during their formation [21]. In our study we observed different localization depending on the species (Fig. S6), suggesting different or multiple roles. Consistent with Waterkeyn and Bienfait we observed a bright calcofluor-fluorescent band between the theca in *N. curvilineata* (Fig. S3c). Additionally, we observed fluorescent strips between each girdle band in most of the species studied. In *N. curvilineata* and *C. cryptica*, we also observed a fluorescent structure associated with the forming valve (Fig. 7). This structure was not observed in the mature valves, suggesting that it is recycled or hidden by the deposition of another layer after valves exocytose. This finding together with the effect of a β-1, 3-glucan synthase inhibitor on the silica structure suggests that in these species the polymer is involved in shaping the valve at the microscale. Many TEM studies reported fibrous structures associated with forming valves, sometimes underlining the SDV and others inhibiting the growth of the silica at a particular location [4]. Some of these could correspond to the glucose polymer we observed, particularly in the raphe and fibulae regions of *N. curvilineata*, where silica deposition was prevented (Fig. 8).

A distinct organic structure containing glucans was observed between the daughter cells during valve formation and released in the medium after separation in *A. salina* and *N. cryptocephala.* These structures could correspond to the transient structure observed by Waterkeyn and Bienfait between the valves of the two daughter cells [21]. The role of this fibrous structure is unclear. The lack of an effect on *A. salina* silica structure after mycafungin treatment is consistent with this material not directly influencing silicification. One hypothesis is that this material participates in the shaping of the protoplast from the outside, or perhaps prevents the two daughter cells from exerting direct pressure on each other. Mucilage secreted between daughter cells has already been observed and suggested to be involved in valve molding [4], [35]. The apparent release of this material into the medium after cell separation (Fig. 7d) suggests that it will contribute to the pool of exopolymeric substances (EPS), which has important consequence for carbon cycling in the oceans.

The presence of organic layers that are fluorescently-labeled only after extraction (Fig. 7, S2), that are only transiently labeled during the process of cell division (Fig. 8), and in which two distinct components are present in the same location in a given species (Fig. S2) confuses their roles. These issues may be reconciled by considering images obtained by Wang *et al*. [36] who applied high-pressure freezing and freeze substitution to TEM thin sections, and observed laminate structures for the diatotepum. This indicates that multiple layers with possible differences in composition could be co-localized in relation to the silica structure (Fig. S6), and that subsequent deposition of one layer could obscure another layer. Different organic layers might be deposited at different times during cell wall formation and only certain layers may be involved in silica structure formation. One question that is not addressed is whether the insoluble organic material is located inside or outside of the SDV membrane.

### Properties of the Insoluble Material

The elasticity determined for the insoluble organic matrix found in the 5 different diatom species studied varied from 6.8 to 11.6 GPa, these values are in the range of the elastic modulus found for natural fibers which vary from 4 to 128 GPa [37] and the cellulosic thecal plate of Dinoflagellates (5 to 12 GPa, [38]). If one of the roles of the internal insoluble matrix is to hold different parts of the frustule together (i.e. valves and girdles bands) high mechanical resistance would be required. The matrix might also be used as a barrier between the plasma membrane and the environment and used by the cell to control its permeability to the external medium. The specific localization of this material in pores is consistent with this. In contrast, structures such as the raphe or ocelli that are involved in secretion of substances remain free of this organic layer.

The proximal organic material is much stiffer than the organic layer on the outside surface of diatom frustules found to range between 0.25 and 0.75 MPa in *P. viridis* and *C. australis* [39] and between 0.1 and 0.5 MPa in the three morphotypes of *P. triconutum* [40]. Our experiments and previous observation [41], show that the surface coating is dissolved by treatment with detergent, which support a different composition and crosslinking comparing this coating with the matrix described in this study. It is possible that the outer surface layer is composed largely of proteins such as the frustulins [42]. However, analysis of the surface of *T. pseudonana* and *P. tricornutum* by X-ray photoelectron spectroscopy, which only interrogates a 10 nm depth, identified abundant amounts of lipid [43]. Reviewing the previous literature suggests the possibility that the organic casing either is, or is associated with, the SDV membrane. Attempts to isolate and purify the SDV membrane have not been conclusive, however with the identification of potential SDV-associated proteins [44] and transgenic approaches, specific marker proteins might be identified that would allow a more definitive conclusion to be reached about the organic casing.

### Conclusions

This work for the first time makes the connection between structure, composition and localization of insoluble organic matrices associated with the diatom cell wall. An important observation of this study is that no insoluble matrix was found encapsulated within the silica in the five diatoms investigated, rather, it was consistently observed on the proximal surfaces of silica structures.

Glucuromannans were found to be the main component of an organic layer localized on the inner surface of the frustule (Fig. S6). This layer probably corresponds to the diatotepum described by Von Stosch [11]. An unresolved question is the extent to which this layer is involved in polymerizing silica or shaping silica structure, which relates to the timing of its appearance relative to silica polymerization, but there is compelling evidence to suggest that it may be involved. Definitive characterization of the roles of these organic materials may require manipulation of the genes encoding or synthesizing their components.

We also confirmed the presence of glucans at different locations in the diatom cell wall (Fig. S6). Interestingly we found that in some species this polymer is involved in valve morphogenesis. Additionally we described a glucose polymer localized between the daughter cells in *A. salina* and *N. cryptocephala*, and released in the medium after cell separation. The species-dependent variation in this layer and examination of other material in different diatom species suggests that the process of structure formation in different species may vary, and that generalization of observations to propose uniform mechanisms of structure formation may be inaccurate. This makes logical sense considering the enormous diversity of structures diatoms are capable of making [26]. We advocate examination of multiple diverse species before establishing general rules.

## Supporting Information

Figure S1 **Different treatments applied to diatom cells and the resulting products.**
(DOCX)

Figure S2 **SDS extracted cell wall of** ***N. curvilineata*** **and** ***C. cryptica*** **stained with DAPI.**
(DOCX)

Figure S3 **Isolated organic matrix from** ***T. dubium*** **.**
(DOCX)

Figure S4 **Calcofluor staining of** ***N. curvilineata*** **(a–d) and** ***C. radiatus*** **(e).**
(DOCX)

Figure S5 **Localization of organic material on the girdle band surfaces of** ***T. pseudonana*** **.**
(DOCX)

Figure S6 **Schematic representation of the putative localization of the mannose-rich (blue) and glucose-rich (green) insoluble matrix associated with the silica (pink) of species examined in this study.**
(DOCX)
