# Supplementary figures and images for: Characterization and Localization of Insoluble Organic Matrices Associated with Diatom Cell Walls: Insight into Their Roles during Cell Wall Formation
Source: PLoS One. 2013 Apr 23;8(4):e61675. doi: 10.1371/journal.pone.0061675 (PMC3633991; doi:10.1371/journal.pone.0061675)

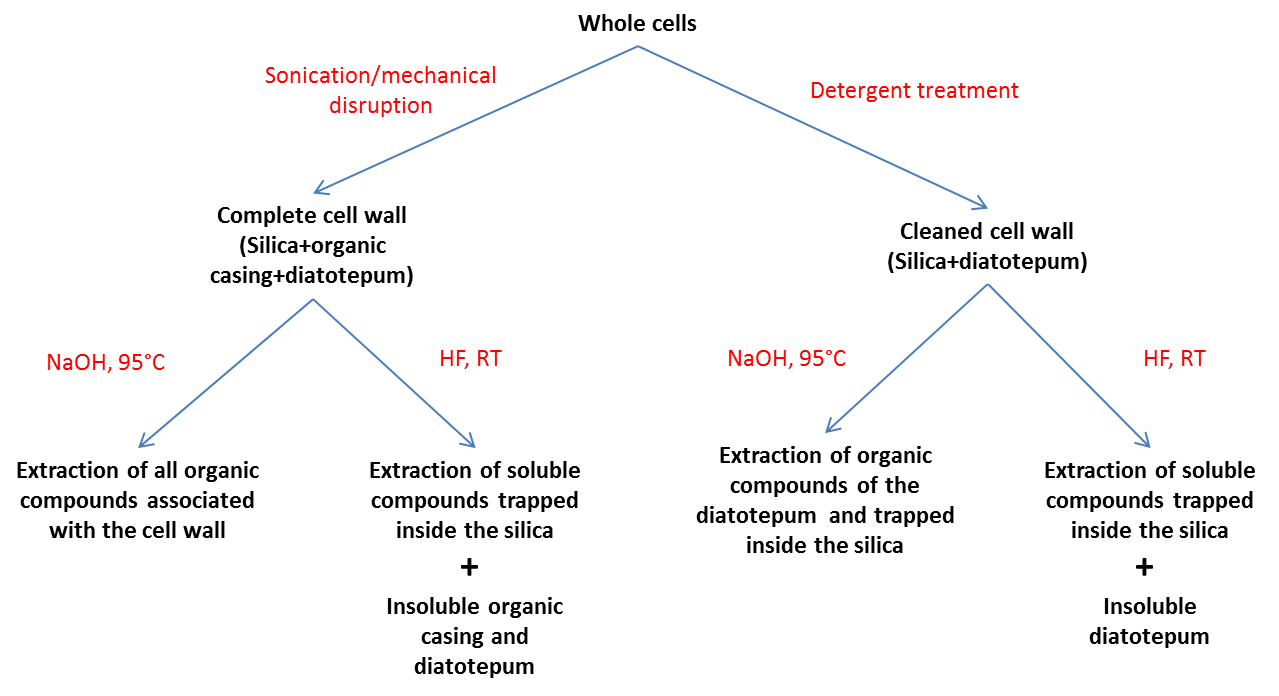


**Figure S1: Different treatments applied to diatom cells and the resulting products.**

Supplement: Figure S1 — Different treatments applied to diatom cells and the resulting products. (DOCX) [file pone.0061675.s001.docx]
