# Supplementary material for: Characterization and Localization of Insoluble Organic Matrices Associated with Diatom Cell Walls: Insight into Their Roles during Cell Wall Formation
Source: PLoS One. 2013 Apr 23;8(4):e61675. doi: 10.1371/journal.pone.0061675 (PMC3633991; doi:10.1371/journal.pone.0061675)
Supplement: Figure S2 — SDS extracted cell wall of N. curvilineata and C. cryptica stained with DAPI. (DOCX) [file pone.0061675.s002.docx]

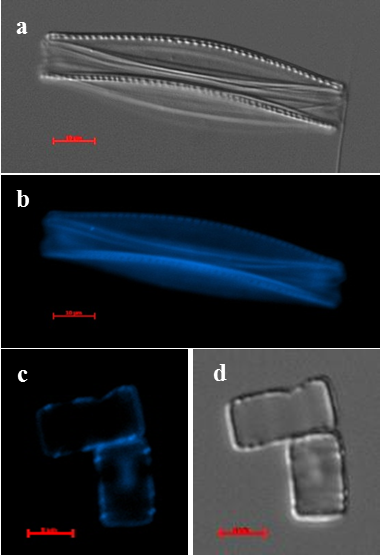


**Figure S2. SDS extracted cell wall of *N. curvilineata* and *C. cryptica* stained with DAPI**. a and d: DIC, c and d: fluorescent micrographs.
