# Supplementary material for: Characterization and Localization of Insoluble Organic Matrices Associated with Diatom Cell Walls: Insight into Their Roles during Cell Wall Formation
Source: PLoS One. 2013 Apr 23;8(4):e61675. doi: 10.1371/journal.pone.0061675 (PMC3633991; doi:10.1371/journal.pone.0061675)
Supplement: Figure S3 — Isolated organic matrix from T. dubium . (DOCX) [file pone.0061675.s003.docx]

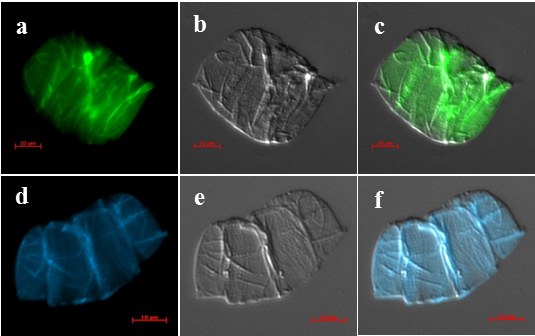


**Figure S3. Isolated organic matrix from *T. dubium*.** a-c: Stained with calcofluor, fluorescent micrograph, DIC and merged respectively. d-f: stained with DAPI. Scale bars=10 µm.
