# Supplementary material for: Characterization and Localization of Insoluble Organic Matrices Associated with Diatom Cell Walls: Insight into Their Roles during Cell Wall Formation
Source: PLoS One. 2013 Apr 23;8(4):e61675. doi: 10.1371/journal.pone.0061675 (PMC3633991; doi:10.1371/journal.pone.0061675)
Supplement: Figure S4 — Calcofluor staining of N. curvilineata (a–d) and C. radiatus (e). (DOCX) [file pone.0061675.s004.docx]

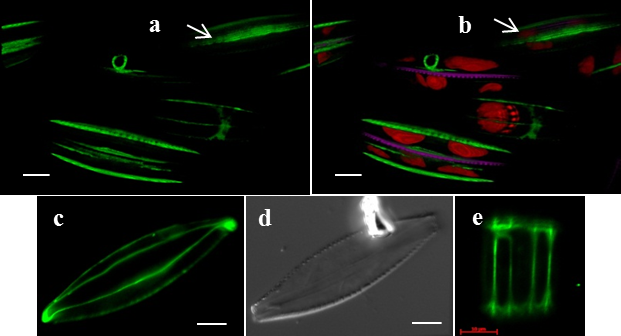


**Figure S4. Calcofluor staining of *N. curvilineata* (a-d) and *C. radiatus* (e).** Fluorescent micrographs show calcofluor stained structure (green), silica (pink) and chlorophyll autofluorescence (red). a, b and e live cells. c and d; SDS extracted cell wall (d=DIC).
