# Supplementary material for: Characterization and Localization of Insoluble Organic Matrices Associated with Diatom Cell Walls: Insight into Their Roles during Cell Wall Formation
Source: PLoS One. 2013 Apr 23;8(4):e61675. doi: 10.1371/journal.pone.0061675 (PMC3633991; doi:10.1371/journal.pone.0061675)
Supplement: Figure S5 — Localization of organic material on the girdle band surfaces of T. pseudonana . (DOCX) [file pone.0061675.s005.docx]

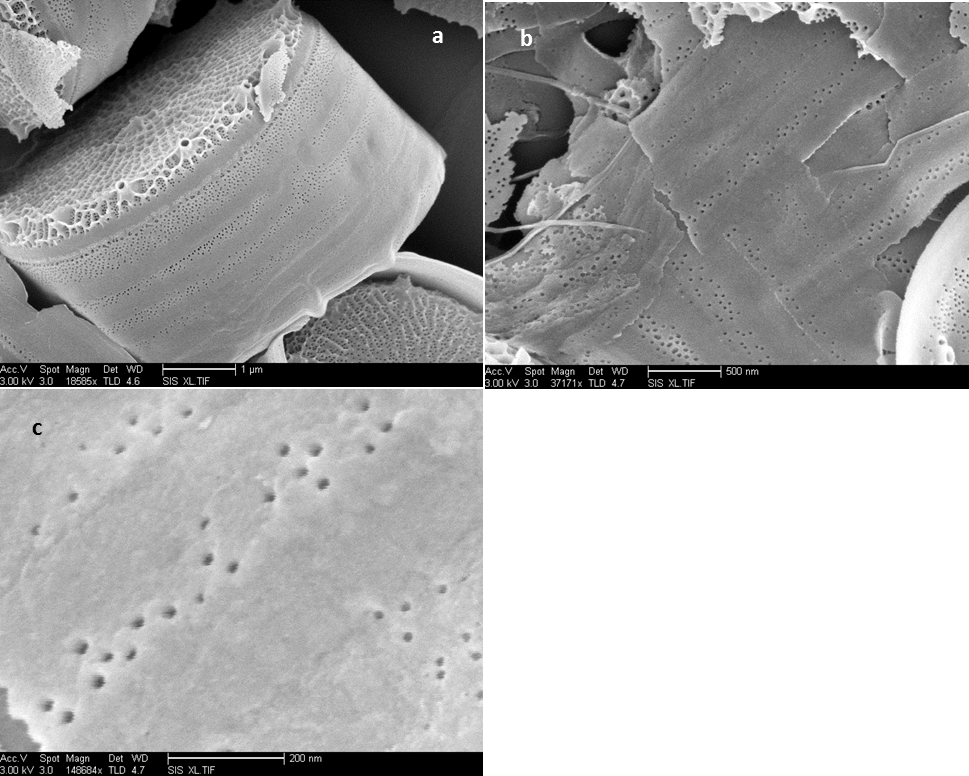


**Figure S5. Localization of organic material on the girdle band surfaces of *T. pseudonana***. SDS cleaned *T. pseudonana*. a: whole cell. b and c: Girdles bands showing organic matter associated with its proximal surface.
