# Supplementary material for: Characterization and Localization of Insoluble Organic Matrices Associated with Diatom Cell Walls: Insight into Their Roles during Cell Wall Formation
Source: PLoS One. 2013 Apr 23;8(4):e61675. doi: 10.1371/journal.pone.0061675 (PMC3633991; doi:10.1371/journal.pone.0061675)
Supplement: Figure S6 — Schematic representation of the putative localization of the mannose-rich (blue) and glucose-rich (green) insoluble matrix associated with the silica (pink) of species examined in this study. (DOCX) [file pone.0061675.s006.docx]

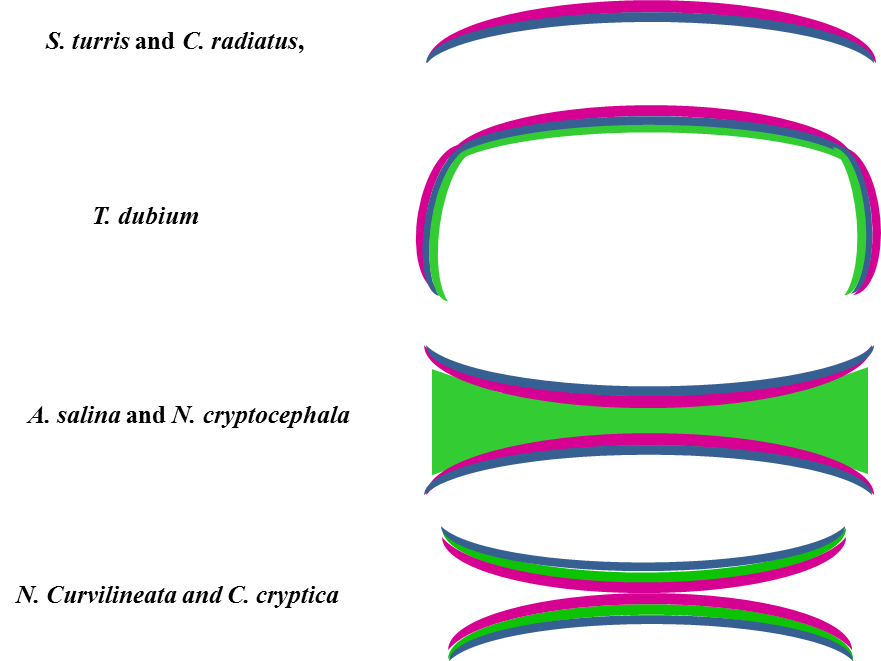


**Figure S6. Schematic representation of the putative localization of the mannose-rich (blue) and glucose-rich (green) insoluble matrix associated with the silica (pink) of species examined in this study.**
